# BEST4⁺ cells: a potential hub of intestinal ion transport and diarrhea manipulation

**DOI:** 10.1007/s44307-026-00126-7

**Published:** 2026-08-12

**Authors:** Hao-zhan Qu, Xiu-qi Wang

**Affiliations:** Agri-Animal Organoid Institute/State Key Laboratory of Swine and Poultry Breeding Industry/Guangdong Provincial Key Laboratory of Animal Nutrition Control/College of Animal Science, South China Agricultural University, Guangzhou, 510642 China

**Keywords:** BEST4⁺ cells, Intestinal epithelium, Ion transport, GC-C/CFTR axis, Secretory diarrhea

## Abstract

BEST4⁺ cells are a recently identified, functionally specialized intestinal epithelial cell population characterized through single-cell and spatial transcriptomic analyses in humans, pigs, rats, and other vertebrates. These cells characteristically express *BEST4*, *OTOP2*, *CA7*, and *GUCY2C*, and exhibit particularly high *CFTR* expression in the small intestine, supporting their roles in luminal pH sensing, fluid-electrolyte homeostasis, and mucus hydration. Emerging evidence from organoid and cross-species studies supports a predominant role for the NOTCH–SPIB signaling axis in driving BEST4⁺ cell differentiation, but their lineage identity and regulatory mechanisms remain incompletely resolved. This review focuses on intestinal epithelial BEST4⁺ cells as a critical cellular hub that connects ion transport physiology with the pathophysiology of secretory diarrhea. We summarize the molecular identity, regional distribution, and cross-species conservation of BEST4⁺ cells. We then evaluate their roles in luminal pH regulation, electrogenic fluid secretion, and mucus barrier integrity. We detail how bacterial enterotoxins activate the GC-C/cGMP/CFTR and cAMP/PKA/CFTR pathways in BEST4⁺ cells to drive pathological fluid hypersecretion, and discuss how viral infections may indirectly engage or amplify BEST4⁺ cell-associated ion-transport pathways. We also outline their relevance to viral diarrhea, inflammatory bowel disease, and cystic fibrosis-associated intestinal dysfunction. Finally, we assess established antidiarrheal approaches and investigational strategies that modulate BEST4⁺ cell-associated pathways, and emphasize the need for cell-type-specific validation in physiologically relevant in vivo models.

## Introduction

The intestinal epithelium consists of finger-like villi projecting into the lumen and crypts of Lieberkühn invaginated into the underlying lamina propria, which together support nutrient absorption and barrier integrity (Neurath et al. 2025). Along the crypt-villus axis, multiple epithelial lineages coexist, including intestinal stem cells, Paneth cells, absorptive enterocytes, microfold (M) cells, goblet cells, enteroendocrine cells, and tuft cells, which collectively maintain epithelial homeostasis and function (Choi and Augenlicht 2024; Clevers 2026). The advent of single-cell RNA sequencing (scRNA-seq) has enabled high-resolution transcriptional profiling of the intestinal epithelium, enabling both the robust identification of canonical epithelial lineages and the discovery of a previously uncharacterized epithelial population defined by a cohesive and distinctive transcriptomic signature, with *bestrophin 4* (*BEST4*) identified as its most highly differentially expressed gene and a lineage-defining transcriptional marker (Parikh et al. 2019). This cell type has since been incorporated into reference atlases of the intestinal epithelium (Elmentaite et al. 2021; Hickey et al. 2023), and accumulating evidence now positions it as a functionally significant, nonredundant component of the epithelial ecosystem, with emerging roles in ion transport, luminal sensing, and barrier regulation.

Research on BEST4 in the intestinal epithelium originated in 2013, when Ito and colleagues (2013) first reported its expression in a spatially restricted subset of absorptive enterocytes using in situ hybridization and immunofluorescence (IF). Since the publication of human intestinal single-cell atlases in 2019, BEST4⁺ cells have been consistently identified across independent studies as a discrete, transcriptionally stable epithelial lineage with conserved anatomical localization and functional signature (Parikh et al. 2019; Burclaff et al. 2022; Malonga et al. 2024). In that landmark study, Parikh and colleagues (2019) characterized BEST4⁺ cells as a mature, stable, and terminally differentiated intestinal epithelial cell type and identified a highly specific gene co-expression signature comprising *BEST4*, *CFTR*, *OTOP2*, *CA7*, *SPIB*, *GUCY2C*, which encodes guanylyl cyclase C (GC-C), and *GUCA2A* and *GUCA2B*, which encode the endogenous ligands of GC-C. Subsequent cross-species investigations have identified morphologically and molecularly conserved BEST4⁺ cells in the porcine small intestine (Wiarda et al. 2023; Xiao et al. 2024; Yu et al. 2025). Although BEST4⁺ cells represent a numerically minor population within the epithelium, their stereotyped co-expression of GC-C, CFTR, CA7, and the acid-sensing channel OTOP2 defines a molecularly coherent and functionally integrated cell type. This signature equips them to act as dedicated luminal pH sensors, bidirectional mediators of transepithelial Cl⁻/HCO₃⁻ transport, and major cellular targets of bacterial enterotoxins, including heat-stable enterotoxin (STa), a high-affinity and selective GC-C agonist, and cholera toxin (CTX), which constitutively activates adenylate cyclase to trigger persistent intracellular cAMP (Wang et al. 2025). Collectively, these advances establish BEST4⁺ cells as physiologically important epithelial effectors that coordinate intestinal pH homeostasis and fluid-electrolyte balance and play a central role in enterotoxin-induced secretory diarrhea.

In this review, we systematically summarize the molecular definition, regional heterogeneity along the intestinal tract and evolutionary conservation across species of BEST4⁺ cells. We also critically evaluate their physiological functions in luminal pH homeostasis, electrogenic fluid secretion, and mucus barrier integrity. We further delineate how bacterial enterotoxins, including STa and CTX, directly exploit the GC-C/cGMP/CFTR and cAMP/PKA/CFTR pathways in BEST4⁺ cells to drive pathological fluid hypersecretion, while discussing how viral infections, including porcine epidemic diarrhea virus (PEDV), rotavirus, and norovirus, may indirectly engage or amplify BEST4⁺ cell-associated ion-transport pathways. We also extend this analysis to their potential involvement in inflammatory bowel disease (IBD) and cystic fibrosis (CF)-associated intestinal dysfunction. Finally, we assess established antidiarrheal approaches and investigational strategies that modulate BEST4⁺ cell-associated pathways, emphasizing the importance of cell-type-specific functional validation in physiologically relevant in vivo contexts.

## Molecular identity of BEST4⁺ cells

### Structural features of BEST4

The *BEST* gene family (*BEST1*–*BEST4*) encodes Ca^2^⁺-activated chloride channels (CaCCs) characterized by a multispanning transmembrane architecture that mediates the selective transport of anions, particularly Cl⁻ and HCO₃⁻, across epithelial membranes (Owji et al. 2021). BEST1 is predominantly expressed in the retinal pigment epithelium and is causally linked to autosomal dominant vitelliform macular dystrophy and other inherited retinopathies. BEST2 is expressed in ocular tissues—including the ciliary body and corneal endothelium—as well as in selected secretory epithelia such as gastric parietal cells and airway submucosal glands (Milenkovic and Weber 2026; Wang et al. 2026a, b; Xiong et al. 2026). BEST3 shows a broader distribution in smooth muscle, cardiomyocytes, and certain non-epithelial stromal compartments (Zhang et al. 2023a, b). In contrast, BEST4 displays exceptional intestinal specificity: it is robustly and selectively expressed in differentiated intestinal epithelium, with the highest abundance in the proximal small intestine and colonic surface epithelium. This distribution makes BEST4 the most intestine-enriched isoform among the bestrophins and a defining molecular feature of a distinct intestinal epithelial lineage (Malonga et al. 2024; Owji et al. 2022).

Recent structural and functional studies demonstrate that bestrophin channels typically assemble as pentameric complexes, with five evolutionarily conserved structural features acting in concert to regulate Ca^2^⁺-dependent gating: (i) an N-terminal Arg-Phe-Pro (RFP) motif essential for pentamer assembly and structural stability; (ii) a hydrophobic “neck” region that functions as the primary Ca^2^⁺-sensitive activation gate; (iii) a cytoplasmic vestibule governing ion selectivity specifically enabling high permeability to Cl⁻ and HCO₃⁻ over other anions; (iv) an acidic calcium-binding domain that transduces transient increases in cytosolic Ca^2^⁺ into conformational changes that open the neck region; and (v) a C-terminal cytoplasmic tail responsible for Ca^2^⁺-dependent inactivation during sustained Ca^2^⁺ elevation or prolonged agonist stimulation (Owji et al. 2022).

**Fig. 1:**
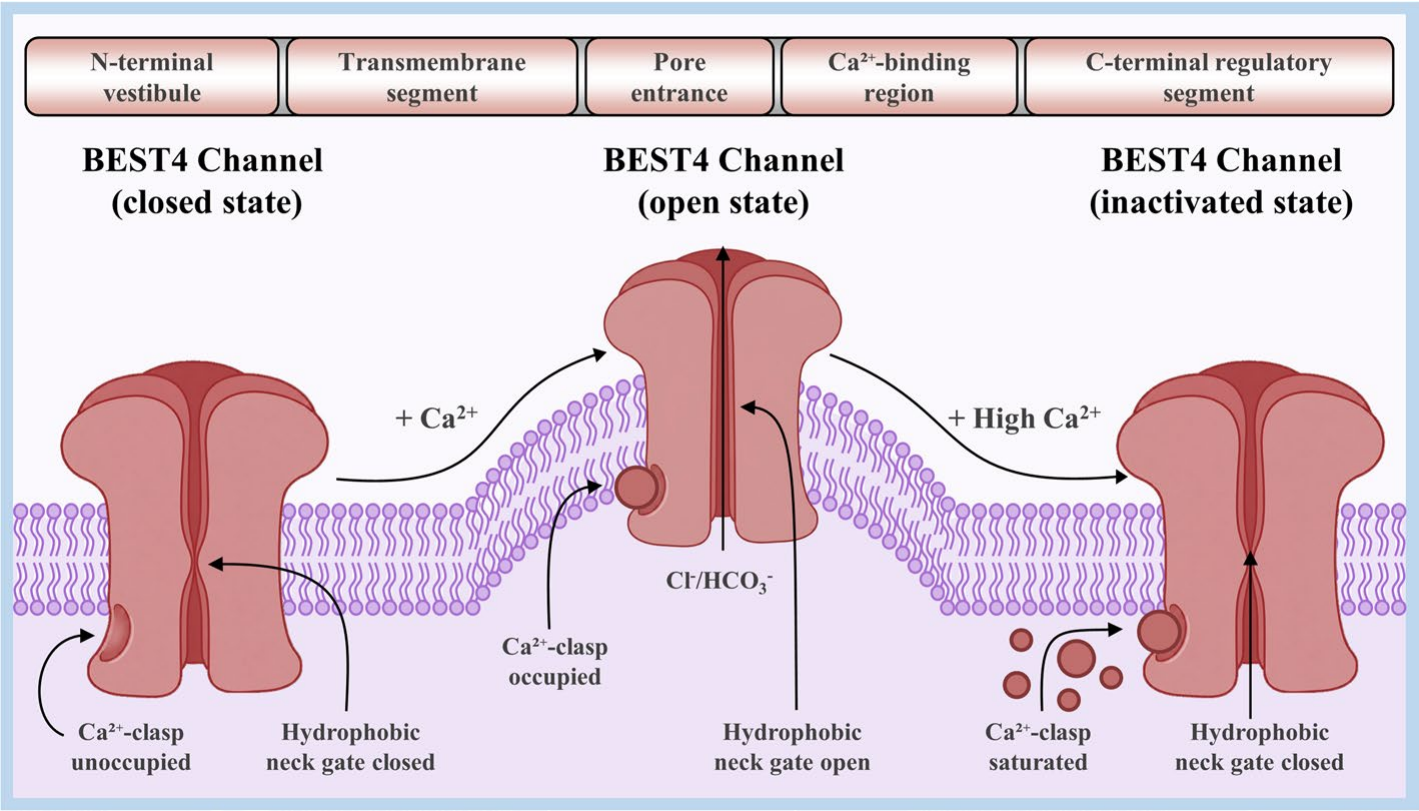
Schematic illustration of the Ca^2^⁺-dependent conformational transitions and gating mechanism of the BEST4 channel. The BEST4 channel consists of the N-terminal vestibule, transmembrane segment, pore entrance, Ca^2^⁺-binding region, and C-terminal regulatory segment. In the resting state, the Ca^2^⁺-clasp is unoccupied and the channel remains closed with the hydrophobic neck gate shut. Upon Ca^2^⁺ binding, the Ca^2^⁺-clasp becomes occupied, the channel opens, and the hydrophobic neck gate opens, allowing Cl⁻/HCO₃⁻ permeation. At high Ca^2^⁺ concentrations, the Ca^2^⁺-clasp becomes saturated, leading the channel into an inactivated state in which the hydrophobic neck gate closes again. The schematic summarizes the transition of the BEST4 channel from the closed state to the open and inactivated states as Ca^2^⁺ concentrations increase

Although high-resolution structural data for BEST4 are scarce relative to those for BEST1 and BEST2, biochemical, electrophysiological, and comparative modeling evidence strongly supports conservation of its pentameric ion-channel architecture and the core functional properties that define the bestrophin family (Owji et al. 2021). Like other bestrophins, BEST4 undergoes Ca^2^⁺-dependent conformational transitions among closed, open, and inactivated states, enabling dynamic, stimulus-responsive regulation of anion flux. This allosteric plasticity likely underpins its physiological role in fine-tuning luminal pH, fluid secretion, and electrolyte homeostasis within the intestinal epithelium (Fig. 1).

### Localization of BEST4⁺ cells

Ito et al. (2013) first reported BEST4 expression in the intestinal epithelium using RNA in situ hybridization and IF; however, the limited spatial resolution of these techniques prevented definitive identification of BEST4⁺ cells as a discrete, terminally differentiated epithelial lineage. Nevertheless, their work provided the foundational evidence for the existence and regional localization of BEST4⁺ cells, which prompted targeted investigations into their developmental origin, molecular regulation, and functional specialization. The advent of single-cell omics has transformed the characterization of BEST4⁺ cells. scRNA-seq remains the gold-standard method for unbiased identification and transcriptional profiling of BEST4⁺ cells within the heterogeneous intestinal epithelium, thereby enabling robust discrimination of functionally distinct subpopulations (Burclaff et al. 2022; Elmentaite et al. 2021; Malonga et al. 2024). Notably, Harnik et al. (2024) integrated spatial transcriptomics, spatial proteomics, and single-molecule fluorescence in situ hybridization to construct a cellular-resolution spatial atlas of the human proximal small intestine. Their work not only localized BEST4⁺ cells with cellular precision but also established a spatial framework for investigating cell–cell paracrine interactions, niche-specific signaling pathways, and functional heterogeneity across anatomical regions.

## Characteristics of intestinal epithelial BEST4⁺ cells

### Development of BEST4⁺ cells

Current evidence suggests that BEST4⁺ cells emerge early in intestinal ontogeny. Comprehensive single-cell transcriptomic profiling has consistently identified BEST4⁺ cells across major developmental stages, from fetal development to childhood and adulthood, demonstrating their persistent presence throughout intestinal development. Notably, BEST4⁺ cells are detectable as early as gestational week 11 in the human fetal intestine, implying lineage specification prior to the completion of villus morphogenesis and functional epithelial maturation. During fetal life, BEST4⁺ cells constitute a rare epithelial population, typically accounting for less than 5% of the intestinal epithelial compartment. In contrast to abundant absorptive enterocytes and canonical secretory lineages such as goblet and Paneth cells, BEST4⁺ cells exhibit a unique molecular signature characterized by BEST4, OTOP2, CA7, CFTR, and GUCY2C expression. This signature is functionally coherent and is consistent with roles in transepithelial anion transport and luminal pH sensing, rather than nutrient absorption, mucus secretion, or immune defense (Elmentaite et al. 2021). The distinguishing molecular, spatial, and functional features of BEST4⁺ cells compared with other major intestinal epithelial cell types are summarized in Table 1. Collectively, these observations support the model that BEST4⁺ cells represent an evolutionarily conserved epithelial subpopulation established early in development that is stably maintained across the lifespan, rather than a postnatally induced or environmentally adaptive cell type.

**Table 1:** Characteristics of BEST4⁺ cells and other intestinal epithelial cells

Cell type	Representative markers	Localization	Major functions
BEST4⁺ cells	*BEST4*, *OTOP2*, *CA7*, *GUCA2A*, *GUCA2B*, *GUCY2C*, *CFTR*, *SPIB*	Small intestine and colon; small-intestinal villus epithelium; colonic crypt apex (Elmentaite et al. 2021; Burclaff et al. 2022; Malonga et al. 2024)	Luminal pH sensing; Cl⁻/HCO₃⁻ transport; mucus hydration (Burclaff et al. 2022; Malonga et al. 2024)
Absorptive enterocytes	*ALPI*, *VIL1*, *SI*, *FABP1*, *APOA4*	Small-intestinal villi (Burclaff et al. 2022; Hickey et al. 2023)	Nutrient absorption; ion and water absorption (Beumer and Clevers 2021; Burclaff et al. 2022)
Colonocytes	*CA1*, *SLC26A2*, *AQP8*, *KRT20*	Colon; upper crypt region (Burclaff et al. 2022; Hickey et al. 2023)	Water and electrolyte absorption; microbial metabolite utilization (Burclaff et al. 2022; Hickey et al. 2023)
Goblet cells	*MUC2*, *SPDEF*, *AGR2*, *CLCA1*, *FCGBP*	Small intestine and colon; relative enrichment in distal intestinal regions; crypt–villus axis (Burclaff et al. 2022; Hickey et al. 2023)	Mucin synthesis; mucus secretion; mucus-layer formation (Gustafsson and Johansson 2022)
Paneth cells	*LYZ*, *DEFA5*, *DEFA6*, *REG3A*	Small intestine; crypt base (Burclaff et al. 2022; Hickey et al. 2023)	Antimicrobial peptide secretion; growth-factor secretion; intestinal stem-cell niche support (Barreto e Barreto et al. 2022; Quintero and Samuelson 2025)
Enteroendocrine cells	*CHGA*, *CHGB*, *NEUROD1*, *INSM1*, *RFX6*	Throughout the intestine; sparse distribution along the crypt–villus axis (Burclaff et al. 2022; Hickey et al. 2023)	Gut hormone secretion; neuropeptide secretion; intestinal function regulation (Atanga et al. 2023)
Microfold (M) cells	*GP2*, *SPIB*, *SOX8*, *ICAM2*, *SLC2A6*, *PTAFR*, *GABRP*	Terminal ileum; follicle-associated epithelium overlying Peyer’s patches (Wang et al. 2026a, b)	Luminal antigen uptake and transepithelial transport; MHC-II-mediated antigen processing and presentation (Ding et al. 2020; Wang et al. 2026a, b)
Tuft cells	*POU2F3*, *TRPM5*, *DCLK1*, *AVIL*	Throughout the intestine; sparse distribution along the crypt–villus axis (Elmentaite et al. 2021; Hickey et al. 2023)	Luminal chemosensing; microbial sensing; activation of type 2 immunity (Feng et al. 2024)
Intestinal stem and progenitor cells	*LGR5*, *OLFM4*, *ASCL2*, *SOX9*, *MKI67*	Throughout the intestine; crypt base; lower crypt; transit-amplifying zone (Beumer and Clevers 2021; Hickey et al. 2023)	Epithelial self-renewal; lineage differentiation; injury-associated regeneration (Beumer and Clevers 2021; Hickey et al. 2023)

The information presented in this table is primarily based on studies of the human intestinal epithelium. The listed markers are representative rather than absolutely cell-type-specific

During intestinal development, BEST4⁺ cells undergo progressive maturation of their functional programs and the establishment of regionally restricted identities. While embryonic BEST4⁺ cells exhibit limited molecular divergence across intestinal segments, pronounced spatial and transcriptional heterogeneity emerges postnatally, particularly in adulthood, resulting in segment-specific differences in localization, gene expression profiles, and functional specialization (Burclaff et al. 2022; Dos Reis et al. 2025). Supporting this developmental trajectory, scRNA-seq analysis of the porcine ileum revealed that the frequency of BEST4⁺ cells remained stable during the physiologically critical transition from lactation to weaning, whereas their transcriptional identity profile progressively acquired regionally appropriate functional features (Tang et al. 2022). This dynamic closely recapitulates the spatiotemporal patterning observed in human intestinal development.

### Regional heterogeneity of BEST4⁺ cells

**Fig. 2:**
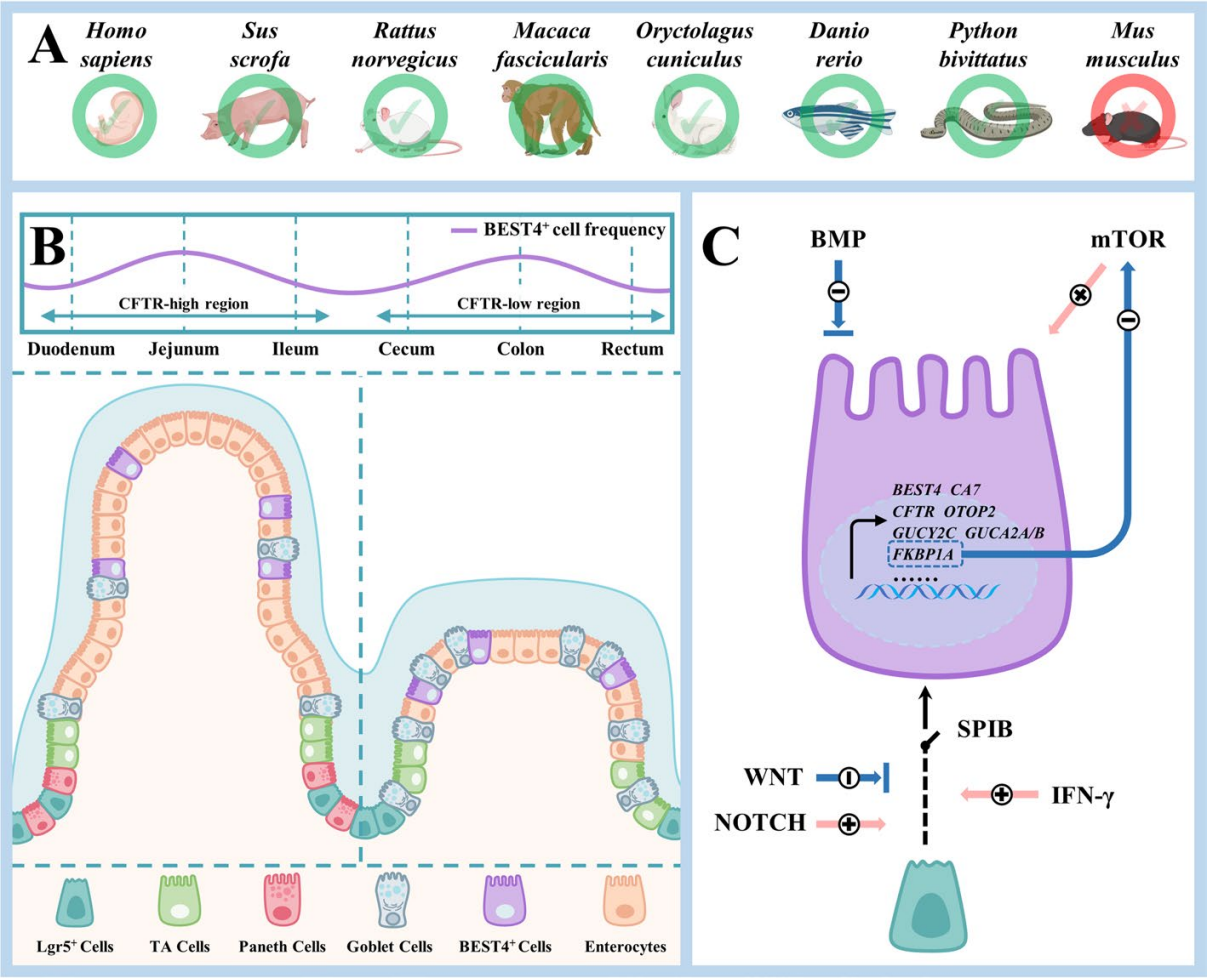
Schematic overview of the cross-species conservation, regional intestinal distribution, and regulatory network of BEST4⁺ cells. **A** BEST4⁺ cells or transcriptionally related epithelial populations have been identified in multiple vertebrate species, including humans (*Homo sapiens*), pigs (*Sus scrofa*), rats (*Rattus norvegicus*), cynomolgus macaques (*Macaca fascicularis*), rabbits (*Oryctolagus cuniculus*), zebrafish (*Danio rerio*), and Burmese pythons (*Python bivittatus*). In contrast, conventional mice (*Mus musculus*) lack a canonical intestinal BEST4⁺ cell population. **B** Schematic representation of regional abundance and epithelial localization of BEST4⁺ cells along the intestine. The upper panel illustrates the relative frequency of BEST4⁺ cells across the duodenum, jejunum, ileum, cecum, colon, and rectum, together with the approximate transition from CFTR-high small-intestinal regions to CFTR-low large-intestinal regions. The lower panel shows the distribution of BEST4⁺ cells along the small-intestinal villus-crypt axis and the colonic crypt–surface axis in relation to other major epithelial cell populations, including LGR5⁺ stem cells, transit-amplifying cells, Paneth cells, goblet cells, and enterocytes. **C** Proposed regulatory model of BEST4⁺ cell differentiation and maintenance. NOTCH signaling promotes BEST4⁺ cell differentiation, whereas WNT and BMP signaling exert inhibitory effects. SPIB functions as a key lineage-associated transcription factor, and IFN-γ may enhance BEST4⁺ cell generation through a SPIB-dependent mechanism. mTOR signaling may support BEST4⁺ cell differentiation or maintenance, whereas FKBP12, the protein encoded by *FKBP1A*, may negatively regulate mTOR activity in mature BEST4⁺ cells as part of a potential feedback mechanism. Representative genes associated with the transcriptional and functional program of BEST4⁺ cells include *BEST4*, *CA7*, *CFTR*, *OTOP2*, *GUCY2C*, *GUCA2A*, *GUCA2B*, and *FKBP1A*. The diagram is schematic and does not represent quantitative cell frequencies or absolute expression boundaries

Although BEST4⁺ cells are distributed throughout the intestine, their abundance and spatial organization exhibit pronounced regional and axial heterogeneity. Integrated analysis of human intestinal single-cell atlases has revealed that BEST4⁺ cell density varies significantly across anatomical segments (Elmentaite et al. 2021). Quantitatively, these cells are comparatively enriched in the jejunum, ileum, and colon, constituting 5%–15% of the epithelial compartment, whereas their proportion is markedly lower in the duodenum and rectum, where they comprise less than 5% of epithelial cells. Along the crypt-villus axis, small-intestinal BEST4⁺ cells are predominantly localized to the upper and middle villus regions, a distribution consistent with roles in rapid transepithelial ion and fluid transport; in contrast, colonic BEST4⁺ cells are concentrated at the crypt apices, directly interfacing with the luminal milieu, suggesting distinct microenvironmental sensing or barrier-modulatory functions (Kim et al. 2025; Fig. 2).

At the functional level, small-intestinal BEST4⁺ cells exhibit robust expression of *CFTR*, consistent with roles in chloride- and bicarbonate-mediated fluid secretion. In contrast, colonic BEST4⁺ cells show elevated expression of *OTOP2* (a proton-selective ion channel) and multiple metallothionein family genes, including *MT1E*, *MT1G*, *MT1H*, *MT1M*, and *MT1X*—molecules implicated in pH buffering, redox homeostasis, and epithelial cytoprotection (Gan et al. 2024; Mitrofanova et al. 2024). Notably, inter-study variability exists regarding the regional enrichment of metallothionein transcripts: while several datasets identify these genes as predominantly enriched in colonic BEST4⁺ cells, others report selective upregulation of specific isoforms, such as *MT1G* and *MT1X*, in subsets of small-intestinal BEST4⁺ cells (Serigado et al. 2022). Furthermore, BEST4⁺ cells show segment-specific expression of antimicrobial peptides: *LYZ* and *DMBT1* are preferentially expressed in the small intestine, whereas *LYPD8*, *WFDC2*, and *SLPI* are enriched in the colon. These spatially resolved molecular signatures indicate that BEST4⁺ cells constitute a functionally adaptable epithelial lineage, retaining a conserved core identity defined by BEST4 expression while acquiring region-tailored effector functions essential for local barrier physiology (Burclaff et al. 2022; Malonga et al. 2024).

### Cross-species distribution of BEST4⁺ cells

BEST4⁺ cells appear to be broadly conserved across vertebrates. Cross-species and species-specific single-cell transcriptomic studies, together with histological validation in several species, have identified BEST4⁺ cells or closely related CA7⁺/OTOP2⁺ epithelial populations in humans (*Homo sapiens*), domestic pigs (*Sus scrofa*), domestic yaks (*Bos grunniens*), European rabbits (*Oryctolagus cuniculus*), Norway rats (*Rattus norvegicus*), cynomolgus macaques *(Macaca fascicularis*), zebrafish (*Danio rerio*), Yangtze sturgeon (*Acipenser dabryanus*), and Burmese pythons (*Python bivittatus*) (Burclaff et al. 2022; Li et al. 2022; Wiarda et al. 2023; Westfall et al. 2024; Chen et al. 2025; Dos Reis et al. 2025; Huang et al. 2026; Malonga et al. 2026; Willms et al. 2026). Despite interspecies variation in spatial distribution, transcriptional identity, and functional specialization, the core molecular signature, including BEST4 itself, exhibits strong evolutionary conservation.

Jakab et al. (2013) identified a subset of CFTR-high enterocytes (CHEs) in the rat small intestinal epithelium using IF labeling. Subsequent scRNA-seq analysis by Zagoren et al. (2025) showed that rat CHEs robustly express *Best4* and share a highly concordant transcriptional signature with human BEST4⁺ cells, establishing them as the functional rodent counterpart of human BEST4⁺ cells. Among non-human models, pigs and cynomolgus macaques exhibit the highest degree of translational relevance: both species harbor clearly definable BEST4⁺ cells whose spatial distribution, marker expression, and functional gene programs closely mirror those observed in humans. Complementary evidence from scRNA-seq studies in rabbits, zebrafish, and additional vertebrate species further supports the deep evolutionary conservation of this epithelial lineage (Li et al. 2022).

However, conventional laboratory mice lack a canonical cellular counterpart to human BEST4⁺ cells. Cross-species genomic and transcriptomic analyses indicate that the murine *Best4-ps* locus is annotated as a pseudogene. Although other genes associated with the human BEST4⁺ cell program, including *Otop2*, *Cftr*, *Guca2a*, and *Guca2b*, remain transcriptionally active in the mouse intestinal epithelium, their expression is distributed across different epithelial populations rather than co-localized within a unified BEST4-defined cell population (Wakisaka et al. 2025). Consequently, conventional mouse models are fundamentally unsuited for mechanistic studies of BEST4⁺ cell biology: they fail to recapitulate not only the canonical marker co-expression but also the integrated physiological functions (such as coordinated ion transport, pH sensing, and luminal defense) that define human BEST4⁺ cells. The intestinal distribution, core molecular markers, and species-specific functional adaptations of BEST4⁺ cells across representative vertebrate species are summarized in Table 2.

**Table 2:** Cross-species comparison of core markers in intestinal BEST4⁺ cells

Species	Intestinal region	Reported gene markers	Major evidence and species-specific features
Human (*Homo sapiens*)	Throughout the intestine	*BEST4*, *OTOP2*, *CA7*, *GUCA2A*, *GUCA2B*, *SPIB*, *GUCY2C*, and *CFTR* (highly expressed in the small intestine)	BEST4⁺ cells represent the most comprehensively characterized reference population. *BEST4*, *OTOP2*, and *CA7* constitute a relatively stable core marker combination, whereas *CFTR* exhibits marked regional specificity and is predominantly enriched in small-intestinal BEST4⁺ cells (Parikh et al. 2019; Elmentaite et al. 2021; Burclaff et al. 2022; Wang et al. 2025)
Pig (*Sus scrofa*)	Duodenum, jejunum, ileum, and cecum	*BEST4*, *OTOP2*, *CA7*, *GUCA2A*, *GUCA2B*, and *CFTR*	Single-cell sequencing and in situ staining have confirmed a distinct intestinal BEST4⁺ cell population. Small-intestinal BEST4⁺ cells are distributed in both the villus and upper-crypt regions and therefore do not fully recapitulate the predominantly villus-associated localization observed in humans (Tang et al. 2022; Li et al. 2022; Wiarda et al. 2023; Xiao et al. 2024)
Cynomolgus macaque (*Macaca fascicularis*)	Ileum	*CA7, OTOP2*, and *GUCA2B* (primarily designated as *CA7*⁺ cells in the available study)	A CA7⁺/OTOP2⁺ cell population corresponding to human and porcine BEST4⁺ cells has been identified in the ileum. However, available evidence is derived mainly from ileal single-cell transcriptomics, and systematic comparisons across intestinal regions are still lacking. This population also shows lower cross-species transcriptional conservation than conventional absorptive enterocytes (Li et al. 2022; Malonga et al. 2024)
Rat (*Rattus norvegicus*)	Duodenum and proximal jejunum	*Best4*, *Otop2*, *Ca7*, *Cftr*, *Gucy2c*, and *Guca2b* (high *Cftr* expression is mainly observed in the proximal small intestine; colonic cells do not exhibit the same *Cftr*-high phenotype)	CHEs in the rat proximal small intestine have been identified as counterparts of human small-intestinal BEST4⁺ cells through transcriptomic and protein-localization analyses. These cells also display molecular features associated with ion transport and luminal pH sensing (Ameen et al. 1995; Jakab et al. 2013; Dos Reis et al. 2025; Zagoren et al. 2025)
Rabbit (*Oryctolagus cuniculus*)	Jejunum, ileum, and cecum	*best4*, *otop2*, *ca7*, *guca2a*, *guca2b*, *spib*, and *cftr* (regionally expressed and not fully concordant with the distribution of *best4*, *otop2*, and *ca7*)	Intestinal cell populations expressing typical *best4*, *otop2*, and *ca7* markers have been identified. However, the core marker combination and *cftr* expression vary according to intestinal region and developmental stage, indicating that their molecular characteristics are not identical to those of human small-intestinal BEST4⁺ cells (Malonga et al. 2024, 2026)
Zebrafish (*Danio rerio*)	Intestine; regional divisions vary among studies	*best4*, *otop2*, *ca7*, and *cftr* (the marker combination varies among developmental stages and datasets)	A distinct best4⁺/otop2⁺ epithelial population has been identified in the zebrafish intestine. These cells retain part of the molecular program associated with ion transport and acid–base homeostasis, although their developmental origin and regulatory mechanisms differ from those of mammalian BEST4⁺ cells (Jones et al. 2023; Sur et al. 2025)
Python (*Python bivittatus*)	Proximal small intestine	*best4* (*otop2*, *ca7*, *cftr*, and *gucy2c* have not been systematically validated)	Single-nucleus RNA sequencing and immunostaining have confirmed the presence of BEST4⁺ epithelial cells in the python proximal small intestine. However, current studies have primarily validated *best4* expression, and the co-expression of other canonical markers remains insufficiently characterized (Westfall et al. 2024)

Only markers explicitly reported in the cited studies are listed. The absence of a reported marker does not necessarily indicate a lack of expression. Marker composition and expression levels may also vary according to intestinal region, developmental stage, physiological condition, and analytical platform

### Experimental models for BEST4⁺ cell research

Selecting an appropriate experimental model is critical for mechanistic dissection and translational validation of BEST4⁺ cell biology. Given that conventional mouse models exhibit negligible or undetectable *Best4* expression in the intestinal epithelium, alternative vertebrate models, particularly species with a conserved BEST4⁺ lineage and relevant intestinal physiology, are essential for addressing current gaps in our understanding of BEST4⁺ cell physiology and pathophysiology.

Rats represent an important small-mammalian model for BEST4⁺ cell research. In addition to harboring a bona fide BEST4⁺ epithelial population, as supported by robust BEST4 protein expression, conserved transcriptional identity, and functional anion transport capacity, rats have a relatively large body size that permits a broad repertoire of physiologically relevant experimental interventions (Ameen et al. 1995; Jakab et al. 2013). Well-characterized rat models of secretory diarrhea, intestinal ion transport dysfunction, and CFTR-related pathophysiology provide physiologically relevant platforms for interrogating BEST4⁺ cells in intact tissue contexts and under defined disease conditions. In addition, rat jejunal organoids robustly generate and stably maintain the BEST4⁺ cell phenotype in vitro (Thiagarajah et al. 2004; Tuggle et al. 2014; Dreano et al. 2019; Zagoren et al. 2023). In the ΔF508-*Cftr* rat model, BEST4⁺ cell abundance increased approximately threefold, accompanied by enhanced apical localization of GC-C (Zagoren et al. 2025; Dos Reis et al. 2025). Nevertheless, critical knowledge gaps persist. First, current mechanistic investigations are predominantly restricted to the duodenum and proximal jejunum; the molecular identity, developmental origin, and functional relationships of distal colonic BEST4⁺ cells remain uncharacterized. Second, rat-specific genetic tools, including BEST4⁺-cell-selective reporter lines, inducible lineage-tracing systems, and conditional knockout models, are still lacking. Consequently, existing conclusions rely primarily on correlative evidence from scRNA-seq profiling, IF colocalization, and pharmacological modulation, with limited direct genetic validation of cell-autonomous mechanisms (Zagoren et al. 2025; Dos Reis et al. 2025).

Pigs are valuable models for both translational medicine and livestock research. The porcine intestine shares important similarities with the human intestine in anatomical structure, nutritional physiology, mucosal development, and epithelial renewal (Mullaney et al. 2022; Schaaf et al. 2023). Genomic comparisons have identified more than 15,000 orthologous genes shared by pigs and humans, with an average amino acid sequence identity of approximately 84.1% among their encoded proteins, indicating considerable conservation of protein-coding genes between the two species (Fang et al. 2012; Warr et al. 2020). Single-cell sequencing has further confirmed the presence of BEST4⁺ cells in the porcine duodenum, proximal jejunum, distal jejunum, and ileum (Tang et al. 2022; Wiarda et al. 2023). However, porcine BEST4⁺ cells are predominantly localized to the upper crypt and lower villus regions, and many genes highly expressed in these cells are also enriched in crypt cells, suggesting that they may retain certain crypt-like molecular features. This distribution differs from that in the human small intestine, where BEST4⁺ cells are located predominantly within the villi rather than the crypts.

Zebrafish also represent a useful non-mammalian model for BEST4⁺ cell research. As a genetically tractable vertebrate with well-established tools for live imaging, CRISPR/Cas9-mediated genome editing, and chemical genetic screening, zebrafish enable high-resolution interrogation of best4-expressing epithelial cell ontogeny, differentiation dynamics, and real-time functional behavior in vivo. Their optical transparency during the larval and juvenile stages, together with rapid ex utero development, facilitates longitudinal tracking of BEST4⁺ cell emergence, spatial patterning, and epithelial integration. However, fundamental interspecies differences in intestinal architecture (e.g., absence of crypt-villus organization), epithelial cell type composition, and luminal microenvironment (e.g., microbiota complexity, bile acid profiles, pH gradients) constrain the direct extrapolation of findings to human intestinal physiology and pathology (Abu-Siniyeh et al. 2025; Willms and Foley 2023; Xia et al. 2022). Consequently, zebrafish are most useful as a discovery platform for conserved developmental principles and core regulatory mechanisms, rather than as a translational surrogate for mammalian models in disease modeling or preclinical therapeutic evaluation.

## BEST4⁺ cell fate commitment

### The lineage identity of BEST4⁺ cells

Whether BEST4⁺ cells belong to the absorptive or secretory lineage remains unresolved. Nevertheless, multiple lines of evidence indicate that they are terminally differentiated epithelial cells: they reside exclusively in post-mitotic compartments (villus tips in the small intestine and apical crypt domains in the colon), spatially segregated from crypt-base proliferative zones, and consistently lack expression of canonical proliferation markers including Ki-67, PCNA, and MCM2 (Burclaff et al. 2022; Harnik et al. 2024; Wakisaka et al. 2025).

Current evidence strongly supports classifying BEST4⁺ cells as a specialized subtype of the absorptive lineage. Single-cell transcriptomics reveals that they co-express canonical absorptive markers, including *VIL1*, *AQP8*, and *SLC26A3*, with mature enterocytes and colonocytes, while simultaneously maintaining a distinct BEST4-associated transcriptional program (Burclaff et al. 2022; Malonga et al. 2024; Parikh et al. 2019). Lineage-tracing studies place BEST4⁺ cells at the terminus of an absorptive differentiation trajectory: they arise from NOTCH-dependent precursors, reside adjacent to mature enterocytes/colonocytes in situ, and exhibit ultrastructural and morphological hallmarks of absorptive epithelia—including microvilli-rich apical surfaces and basolateral ion transporter localization—confirmed by confocal and electron microscopy (Wakisaka et al. 2025). Mechanistically, NOTCH signaling directly governs their differentiation: human organoids require NOTCH activation for BEST4⁺ cell emergence, and these cells robustly express the NOTCH2 receptor. Critically, Wang et al. (2024) demonstrated via ChIP-qPCR and luciferase reporter assays that the NOTCH effector HES4 binds a functional enhancer in the *BEST4* 5′ regulatory region and potently activates its transcription—establishing a direct, evolutionarily conserved NOTCH–HES4–BEST4 axis. Collectively, multimodal evidence across human tissues, organoids, and disease models positions BEST4⁺ cells as a functionally specialized, terminally differentiated branch of the absorptive lineage.

Conversely, emerging evidence links BEST4⁺ cells to the secretory lineage. Burclaff et al. (2022) integrated scRNA-seq with pseudotime trajectory inference across the healthy adult human small intestine and colon, and identified a low-probability but statistically significant developmental branch connecting BEST4⁺ cells to ATOH1⁺ secretory progenitors, accompanied by coordinated upregulation of secretory-associated genes including NPY and BMP3, with NPY expression rising progressively during BEST4⁺ cell maturation. In zebrafish, direct in vivo lineage tracing demonstrates that BEST4⁺ cells derive exclusively from ATOH1⁺ secretory precursors, and NOTCH/Dll4 signaling actively biases cell fate toward either the BEST4⁺ or enterochromaffin lineage (Sur et al. 2025). This apparent divergence from the absorptive model likely reflects genuine biological heterogeneity—not a methodological artifact—arising from species-specific developmental programs, regional epithelial specialization (e.g., duodenal vs. colonic), or context-dependent plasticity under homeostatic versus inflammatory conditions.

### NOTCH–SPIB axis in BEST4⁺ differentiation

As a terminally differentiated epithelial lineage derived from LGR5⁺ intestinal stem cells, BEST4⁺ cell fate is stringently governed by the NOTCH signaling pathway (Malonga et al. 2024). Beyond the established roles of NOTCH2 receptor expression and HES4-mediated transcriptional activation of *BEST4*, the transcription factor SPIB functions as a nonredundant, lineage-determining regulator. CRISPR–Cas9–mediated *SPIB* knockout in human small-intestinal organoids under conditions in which NOTCH signaling remained intact abolished BEST4⁺ cell generation, demonstrating an absolute requirement for SPIB in lineage commitment. In contrast, *SPIB* overexpression failed to expand the BEST4⁺ population, confirming that SPIB is necessary but insufficient for terminal differentiation (Wang et al. 2025). Furthermore, snRNA-seq and ligand–receptor mapping in the python small intestine revealed that BEST4⁺ cells autonomously express NOTCH ligands, while adjacent immune and endothelial cells show high NOTCH1 expression, indicating a local juxtacrine NOTCH signaling niche (Westfall et al. 2024).

Mechanistically, this finding aligns with established principles of intestinal epithelial lineage specification. In the canonical NOTCH signaling paradigm, in which ligand-induced NOTCH activation drives stem cell commitment toward the absorptive lineage, BEST4⁺ cell differentiation represents a specialized terminal branch (Faizo 2024; Kolev and Kaestner 2023; Yang et al. 2025). Here, NOTCH signaling establishes a permissive transcriptional landscape for BEST4⁺ fate acquisition, while SPIB functions as the indispensable, lineage-restricted executor that directly activates *BEST4* and co-regulates key functional genes, including CA7, CFTR, and other lineage-associated factors (Fig. 2).

Notably, downstream effectors show evolutionary divergence: in zebrafish, meis1b, not SPIB, serves as the critical NOTCH-dependent transcription factor specifying BEST4⁺ identity (Sur et al. 2025). This species-specific substitution underscores that, although the upstream regulatory logic is conserved, the terminal effector module has undergone functional rewiring—highlighting SPIB’s nonredundant role in mammalian BEST4⁺ cell specification.

### Other regulatory pathways

Beyond the core NOTCH–SPIB axis, WNT, BMP, and mTOR signaling pathways modulate BEST4⁺ cell differentiation in a context-dependent and often antagonistic manner. Human intestinal organoid studies demonstrate that canonical WNT and BMP signaling act as potent suppressors of BEST4⁺ cell commitment: exogenous WNT3A or BMP2/4 treatment significantly reduces BEST4⁺ cell frequency, whereas inhibition of BMP signaling with Noggin enhances their generation. Conversely, mTORC1 activation promotes BEST4⁺ cell emergence and maintenance—as indicated by an increased abundance of BEST4⁺ cells upon treatment with the mTOR activator MHY-1485 and diminished formation following rapamycin-mediated mTORC1 inhibition (Wang et al. 2025). Notably, mature BEST4⁺ cells may also express high levels of FKBP12, which is encoded by FKBP1A and binds rapamycin, thereby conferring rapamycin sensitivity. This expression pattern supports a stage-dependent regulatory model: mTORC1 activity is required for early-to-intermediate differentiation steps, while FKBP12–mediated feedback likely constrains mTORC1 signaling to consolidate terminal identity and ensure functional stability (Fig. 2). Importantly, these mechanistic insights derive exclusively from in vitro organoid systems; their physiological relevance to native crypt-villus architecture, dynamic cell turnover, and stromal–epithelial crosstalk has not yet been validated in vivo.

Cytokines in the immune microenvironment may actively modulate BEST4⁺ cell differentiation. Human organoid studies demonstrate that IL-22, IL-17A, IL-25, and IFN-γ increase BEST4⁺ cell frequency—with IFN-γ exerting the strongest effect—by accelerating differentiation rather than promoting proliferation of mature cells. Critically, this IFN-γ–driven expansion is SPIB–dependent: genetic ablation of *SPIB* abolishes the response, confirming that cytokine signaling converges on the core NOTCH–SPIB transcriptional axis to specify BEST4⁺ fate. While IFN-γ receptor subunits (IFNGR1/IFNGR2) are expressed on intestinal stem and transit-amplifying cells, whether direct ligand–receptor engagement is the primary mechanism remains unclear. Complementary evidence shows that IL-4, IL-6, TNF-α, and RANKL suppress BEST4⁺ cell generation, establishing a cytokine-mediated regulatory network with opposing effects that fine-tunes epithelial lineage output in response to immune cues (Wang et al. 2025).

## Physiological functions of BEST4⁺ cells

### Ion transport and fluid-electrolyte homeostasis

Molecular profiling of small-intestinal BEST4⁺ cells supports a primary role for these cells in intestinal ion transport and fluid-electrolyte homeostasis. *BEST4*, the defining marker of this lineage, functions as a Ca^2^⁺-activated anion channel. Structural and functional homology with *BEST1*–*BEST3* supports its capacity to mediate transmembrane Cl⁻ and HCO₃⁻ conductance upon cytosolic Ca^2^⁺ elevation (Owji et al. 2021, 2022). CFTR serves as the principal effector channel, mediating the apical secretion of these anions into the intestinal lumen. Upstream of CFTR, the GC-C/cGMP axis constitutes a dedicated autocrine signaling module that tonically activates CFTR; guanylin and uroguanylin bind and activate the receptor GC-C, triggering cGMP production and subsequent PKG-mediated CFTR phosphorylation (Keely and Barrett 2022; Prasad et al. 2022; Sur et al. 2025; Tümmler et al. 2025; Wang et al. 2025; Yuan et al. 2025). Critically, BEST4⁺ cells co-express all components of this module (including ligands, receptor, and effector) at high levels, strongly supporting autonomous, self-sustaining ion secretion. These molecular–functional relationships have been experimentally recapitulated in human intestinal organoids, confirming their biochemical plausibility; however, direct electrophysiological validation of BEST4 channel activity and demonstration of coordinated BEST4/CFTR function in native tissue or in vivo models are still needed.

### Acid–base sensing and pH regulation

In BEST4⁺ cells, BEST4, GC-C, and CFTR form a coordinated functional triad that mediates luminal HCO₃⁻ secretion and actively maintains local pH homeostasis in the intestinal lumen. In addition, OTOP2, a proton-selective ion channel with broad pH sensitivity (pH 5–10), functions as an extracellular pH sensor and proton-conducting effector. Under acidic extracellular conditions, OTOP2 mediates inward proton flux, whereas, under neutral-to-alkaline extracellular conditions, it can mediate outward proton currents, indicating bidirectional proton conduction determined by the electrochemical gradient (Teng et al. 2022). Notably, in rat small-intestinal BEST4⁺ cells, luminal acidification triggers rapid apical enrichment of OTOP2, GC-C, and CFTR—spatially coupling pH sensing to HCO₃⁻ secretion (Dos Reis et al. 2025). This colocalization, combined with the relative rarity of proton-selective channels in mammalian epithelia, strongly supports a model in which BEST4⁺ cells function as integrated pH-sensing and pH-regulating units, dynamically adjusting luminal acidity through coordinated OTOP2–GC-C–CFTR activity.

### Mucus hydration and barrier homeostasis

**Fig. 3:**
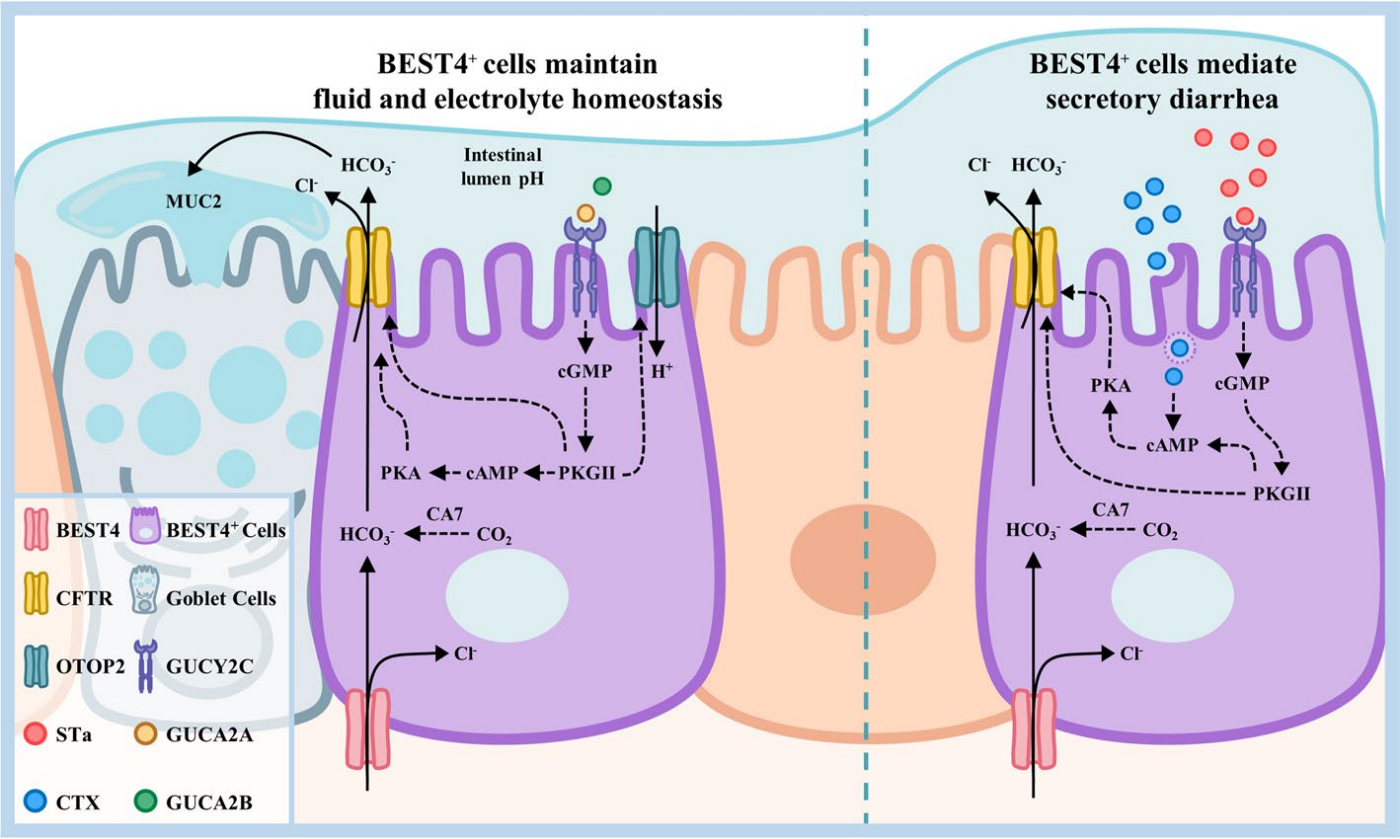
Proposed physiological and pathophysiological roles of intestinal BEST4⁺ cells. Under homeostatic conditions, BEST4⁺ cells contribute to fluid and electrolyte homeostasis through a gene-expression program characterized by *BEST4*, *CFTR*, *OTOP2*, *CA7*, and *GUCY2C*. At the protein level, OTOP2 may sense luminal acidity, CA7 supports intracellular HCO₃⁻ generation, and BEST4 and CFTR mediate Cl⁻ and HCO₃⁻ transport, thereby contributing to local pH regulation, fluid secretion, and mucus hydration in coordination with goblet cells. The endogenous GC-C ligands guanylin and uroguanylin, encoded by *GUCA2A* and *GUCA2B*, respectively, may further promote this secretory program through cGMP-dependent signaling. Under pathological conditions, BEST4⁺ cells may serve as important epithelial effector cells in secretory diarrhea because they are enriched in components of the GC-C/cGMP/CFTR signaling axis. STa activates the GC-C/cGMP/PKG pathway, whereas CTX activates the cAMP/PKA pathway; both pathways converge on CFTR activation, thereby enhancing Cl⁻ and HCO₃⁻ secretion, increasing luminal water accumulation, and ultimately promoting diarrhea

The intestinal mucus barrier is largely formed by goblet cell-derived MUC2 (Du et al. 2026; Wu et al. 2026). Upon exocytotic release into the lumen, highly concentrated MUC2 undergoes rapid hydration and expansion, a process critically dependent on luminal HCO₃⁻-mediated neutralization of the acidic microenvironment proximal to the epithelium (Baird 2026; Qiao et al. 2025; Song et al. 2023). Given their close anatomical proximity to goblet cells in the human small intestine, BEST4⁺ cells are strategically positioned to supply this essential HCO₃⁻ flux, functioning as a dedicated paracrine source for mucus barrier assembly (Ljungholm et al. 2024; Stanforth et al. 2024; Yang et al. 2013; Fig. 3). This proposed role is supported by their co-expression of CFTR (the principal HCO₃⁻ secretory channel) and OTOP2 (a rapid pH sensor that may dynamically tune HCO₃⁻ secretion in response to luminal acidification). Supporting this model, Gustafsson et al. (2015) demonstrated that CFTR-dependent HCO₃⁻ secretion is indispensable for mucin release and barrier integrity: *Cftr*^⁻^/^⁻^ mice exhibit viscous, adherent ileal mucus, whereas exogenous NaHCO₃ fully rescues mucus hydration and detachment. While the spatial and molecular evidence strongly implicates BEST4⁺ cells in this process, direct evidence that these cells supply HCO₃⁻ for mucus biogenesis remains lacking, as does causal validation through lineage-specific ablation or inhibition.

## BEST4⁺ cells and intestinal disease

### Secretory diarrhea

Diarrhea is commonly classified into four mechanistic categories—osmotic, exudative, motility-related, and secretory—each defined by distinct underlying mechanisms rather than merely symptomatic presentation. Osmotic diarrhea arises from intraluminal accumulation of non-absorbable solutes (e.g., undigested disaccharides or polyethylene glycol), which establishes an osmotic gradient that draws water osmotically across the epithelium into the lumen. Exudative diarrhea reflects active mucosal injury: inflammatory infiltration, epithelial desquamation, and ulceration compromise barrier integrity, permitting unregulated efflux of plasma proteins, mucus, neutrophils, erythrocytes, and cellular debris into the lumen. Motility-related diarrhea results from accelerated propulsive motility, drastically reducing intestinal transit time and thereby truncating the critical period required for efficient absorption of water and electrolytes. Secretory diarrhea, in contrast, is fundamentally an epithelial transport disorder, characterized by net luminal secretion of Cl⁻ and HCO₃⁻ that exceeds absorptive capacity. It results not from altered transit or barrier loss but from dysregulated signaling cascades that directly modulate ion channel activity. Specifically, hyperactivation of the GC-C/cGMP/CFTR and cAMP/PKA/CFTR axes drives excessive CFTR-dependent anion secretion while concurrently suppressing key absorptive transporters such as NHE3 and H⁺/K⁺-ATPase (Marasco et al. 2026; Keely and Barrett 2022). BEST4⁺ cells are uniquely positioned to amplify this pathology: they co-express the full complement of pathway components—including GC-C, transmembrane and soluble adenylate cyclases (ADCY3, ADCY9, and sAC), PKG/PKA, and CFTR—at functionally relevant levels, establishing them as central effectors in secretory diarrhea pathogenesis. Physiologically, CFTR-mediated Cl⁻ and HCO₃⁻ secretion maintains luminal pH homeostasis and enables MUC2 mucin expansion through localized alkalinization—thereby underpinning mucus barrier architecture and function (Amiri et al. 2026). Under pathological stimulation, such as exposure to heat-stable enterotoxin STa or TNF-α, the same molecular machinery can become excessively activated, inducing sustained CFTR membrane insertion and prolonged channel open probability. This shifts the epithelium from a net absorptive to a net secretory state, generating an osmotic force sufficient to drive substantial net water flux into the lumen and precipitating profuse watery diarrhea.

#### Bacterial diarrhea

Although both BEST4⁺ cells and enteroendocrine cells express high levels of GC-C, enteroendocrine cells exhibit negligible or undetectable CFTR protein, rendering BEST4⁺ cells the sole intestinal epithelial population in humans that co-expresses GC-C and CFTR at functionally significant levels (Wang et al. 2025). This unique molecular signature positions BEST4⁺ cells as major epithelial effectors of secretory diarrhea. Mechanistically, STa induces sustained cGMP-dependent CFTR opening in human intestinal organoids by binding to and hyperactivating GC-C, resulting in robust anion secretion, osmotic water influx, and characteristic organoid swelling (Triana et al. 2021; Wang et al. 2025; Yuan et al. 2025). Similarly, CTX activates CFTR in BEST4⁺ cells via ADP-ribosylation of Gαs, leading to persistent cAMP accumulation, PKA-mediated CFTR phosphorylation, and prolonged channel activity. Notably, *enterotoxigenic Escherichia coli* (*ETEC*), a major enteric pathogen in swine production, exploits this same molecular vulnerability: its STa engages the GC-C/cGMP/CFTR axis, while its heat-labile enterotoxin LT mimics CTX by constitutively activating adenylate cyclase and the cAMP/PKA/CFTR pathway (Motyka et al. 2022; Read et al. 2014; Sheikh et al. 2022; Wang et al. 2025; Yuan et al. 2025; Zhang and Sack 2025). Despite compelling in vitro and mechanistic evidence, direct in vivo validation, particularly in porcine models of *ETEC*-induced secretory diarrhea, is still needed to determine whether BEST4⁺ cells are the dominant cellular targets mediating toxin-driven fluid loss.

#### BEST4⁺ cells in viral diarrhea

Enteric viruses, including PEDV, rotavirus, and norovirus, are major causes of acute watery diarrhea in humans and livestock. Whether BEST4⁺ cells are direct targets of these viruses remains unknown. Nevertheless, their expression of ion-transport and pH-regulating molecules, including BEST4, CFTR, CA7, OTOP2, and GC-C, suggests that they may act as secondary epithelial responders that modulate ion secretion, luminal pH, and mucus hydration during infection.

PEDV preferentially infects and destroys villus absorptive enterocytes, triggering villus atrophy, malabsorption, and impaired nutrient uptake. Its S1 spike protein activates EGFR-ERK signaling, leading to transcriptional repression and impaired membrane trafficking of the Na⁺/H⁺ exchanger NHE3—thereby diminishing Na⁺-coupled water absorption (Zhang et al. 2023a, b). Consistent with this, PEDV-infected piglets exhibit reduced transcript abundance of *AQP3*, *AQP8*, *AQP10*, and *NHE3* in the jejunum and ileum, coupled with increased ileal *CFTR* transcript levels (Zhang et al. 2019). Electrophysiological analyses further reveal enhanced cAMP-dependent electrogenic secretion in jejunal tissues, as indicated by enhanced short-circuit current responses to isoproterenol and forskolin/IBMX (Enns et al. 2018). Notably, while jejunal *CFTR* mRNA remains unchanged, the Ca^2^⁺-activated K⁺ channel KCNN4 and the Ca^2^⁺-activated Cl⁻ channel TMEM16A are upregulated—potentially augmenting the electrochemical gradient for Cl^⁻^ efflux. Given that porcine small-intestinal BEST4⁺ cells robustly express CFTR (Wiarda et al. 2023), they represent a plausible cellular source of virus-associated anion secretion. The combined suppression of NHE3-mediated absorption and maintained or enhanced BEST4/CFTR/CA7-mediated anion secretion likely drives a net shift toward fluid secretion. However, direct lineage-tracing or cell-type-specific functional evidence confirming the contribution of BEST4⁺ cells to these secretory changes is lacking.

Rotavirus induces diarrhea through a distinct multicomponent mechanism: its nonstructural protein NSP4 disrupts intracellular Ca^2^⁺ homeostasis in infected cells, which triggers ADP release, paracrine activation of P2Y1 receptors on adjacent uninfected epithelial cells, and propagation of intercellular Ca^2^⁺ waves (Chang-Graham et al. 2020). BEST4⁺ cells located near infection foci may respond to this Ca^2^⁺ signal via BEST4-dependent Cl⁻/HCO₃⁻ secretion, functioning as “bystander effectors” in fluid loss. In contrast, CFTR involvement has not been established, because neither the cAMP nor the cGMP pathway has been shown to be activated during rotavirus infection. Additionally, NSP4 stimulates enterochromaffin cells to secrete serotonin, thereby engaging enteric and vagal neural circuits that regulate secretion, motility, and emesis (Hagbom et al. 2011). While BEST4⁺ cells extend neuropod-like basal processes and reside in close proximity to enteric neurons (Dos Reis et al. 2025), their participation in serotonin-mediated neuroepithelial signaling remains speculative.

Norovirus infection compromises absorptive function, increases paracellular permeability, and induces sustained electrogenic anion secretion. Human duodenal biopsies show reduced transepithelial electrical resistance, elevated basal short-circuit current, and heightened Cl⁻ secretion (Troeger et al. 2009). Norovirus can replicate productively in differentiated enterocytes and subsets of enteroendocrine cells (Ettayebi et al. 2016; Green et al. 2020); however, whether mature BEST4⁺ cells support viral replication—or contribute to the observed secretory phenotype via BEST4, CFTR, TMEM16A, or other anion transporters—remains unknown.

Viral inflammation may also reshape BEST4⁺ cell dynamics. IFN-γ, a hallmark cytokine of antiviral immunity, drives BEST4⁺ cell differentiation in human intestinal organoids through a SPIB-dependent transcriptional program; these IFN-γ-primed cells exhibit markedly enhanced CFTR-dependent fluid secretion upon subsequent toxin challenge (Wang et al. 2025). Thus, virus-induced IFN-γ production could expand the BEST4⁺ cell pool and potentiate their secretory, pH-regulatory, and mucus-modulating functions. The effects of this modulation may be stage dependent: during acute infection, BEST4⁺-driven hypersecretion may exacerbate dehydration, whereas during the resolution phase, HCO₃⁻-dependent mucus hydration and barrier reinforcement may facilitate epithelial repair. To date, no in vivo studies have confirmed IFN-γ-mediated expansion of BEST4⁺ cells in viral enteritis.

### Inflammatory bowel disease (IBD)

Parikh et al. (2019) provided foundational evidence linking BEST4⁺ cells to IBD pathogenesis. In human colonic tissue from IBD patients, BEST4⁺ cells showed a significant reduction in abundance and dysregulated expression of 28 genes—including marked downregulation of metallothionein family members (*MT1M*, *MT1H*, *MT1F*, *MT1E*) and key ion transport effectors (*SLC26A2*, *CA1*, *FABP1*, *SELENBP1*). These transcriptional alterations indicate that IBD compromises both metal-ion buffering capacity and epithelial ion transport function specifically within the BEST4⁺ lineage. Although the magnitude of transcriptional change in BEST4⁺ cells is less pronounced than in bulk colonocytes, their selective vulnerability, reflected in the consistent dysregulation of metal-handling and transport pathways, suggests a nonredundant role in epithelial stress responses during chronic inflammation.

### Cystic fibrosis (CF)

The role of BEST4⁺ cells in CF remains incompletely characterized, although emerging evidence points to their pathophysiological relevance. CF is an autosomal recessive disorder caused by loss-of-function mutations in *CFTR*, leading to defective transepithelial Cl⁻ and HCO₃⁻ secretion—particularly in the intestine, where impaired anion transport results in dehydrated, hyperconcentrated mucus and compromised mucus clearance (Burton et al. 2021; Ota et al. 2026; Pawłowska et al. 2025). Within the intestinal epithelium, BEST4⁺ cells constitute a functionally distinct subpopulation and express exceptionally high levels of CFTR transcripts, making them among the most CFTR-dependent epithelial cells and thus highly vulnerable to *CFTR* mutation-associated dysfunction (Bulcaen et al. 2024; Knoll et al. 2025). Consistent with this, Dos Reis et al. (2025) demonstrated in a ΔF508-*Cftr* rat model of CF that while BEST4⁺ cell numbers increased, apical membrane localization of CFTR protein was severely disrupted—a defect directly linked to the canonical trafficking failure of mutant CFTR. This mislocalization likely impairs BEST4⁺-mediated anion secretion, pH sensing, and mucus hydration, thereby contributing to the hallmark intestinal manifestations of CF, including luminal acidosis and viscous mucus accumulation. Notably, the same study reported compensatory upregulation of GC-C at the apical membrane of BEST4⁺ cells in ΔF508-*Cftr* rats—an adaptive response potentially aimed at restoring cGMP-dependent signaling and residual CFTR activity, though it may also exacerbate secretory dysregulation under pathological conditions. Collectively, these findings position BEST4⁺ cells not as passive bystanders but as active contributors to CF-associated intestinal ion transport failure, luminal pH dysregulation, and mucus barrier pathology.

## Targeted modulation of intestinal epithelial BEST4⁺ cells

### Targeting the GC-C/cGMP/CFTR axis

Targeting the GC-C/cGMP/CFTR axis, which is enriched in BEST4⁺ cells, represents a mechanistically sound strategy for controlling excessive intestinal secretion. The most direct strategy is to inhibit GC-C. In patient-derived intestinal organoids carrying activating *GUCY2C* mutations, the GC-C-specific inhibitor SSP2518 reduced intracellular cGMP levels and effectively blocked CFTR-dependent chloride secretion induced by STa, providing experimental support for targeting this pathway to treat GC-C-associated diarrhea (van Vugt et al. 2021).

However, complete or sustained GC-C blockade is undesirable. Physiological guanylin/uroguanylin-GC-C signaling is nonredundant for fluid and electrolyte homeostasis, mucus hydration, epithelial barrier integrity, and turnover. GC-C-deficient mice display baseline jejunal paracellular hyperpermeability, tight junction disassembly (JAM-A, claudin-2), and elevated myosin light chain phosphorylation—hallmarks of cytoskeletal-driven barrier failure (Han et al. 2011). Following immune challenge with lipopolysaccharide (LPS) or enteric infection (*Salmonella enterica serovar Typhimurium*), these defects escalate into systemic bacterial translocation, multiorgan bacterial burden, severe inflammation, and markedly reduced survival (Han et al. 2011; Amarachintha et al. 2018). These findings indicate that GC-C functions not merely as a modulator but also as a guardian of mucosal defense, rendering global suppression clinically untenable. Therapeutic success therefore hinges on selectively silencing *pathological* overactivation while preserving basal tone. This may be achieved through gut-restricted, transient, or functionally biased modulation.

As a key downstream effector in this pathway, CFTR represents another critical target. CFTRinh-172 and GlyH-101 are widely used experimental CFTR inhibitors to validate antisecretory effects. In mouse models, a single intraperitoneal injection of CFTRinh-172 reduces CTX-induced jejunal fluid secretion by more than 90%, whereas intraluminal administration of GlyH-101 decreases intestinal luminal fluid accumulation by approximately 80% in closed-loop models (Ma et al. 2002; Muanprasat et al. 2004). Cryo-EM studies reveal that CFTRinh-172 binds within the intracellular pore region of CFTR, stabilizing its inactive conformation, thereby providing structural insights for rational design of highly selective inhibitors (Young et al. 2024; Gao et al. 2024). However, clinical translation of these compounds is limited by narrow therapeutic windows, difficulty in controlling in vivo exposure, and restricted routes of administration. In vitro, 24-h exposure to CFTRinh-172 or GlyH-101 at concentrations of 1–20 μM did not significantly affect cell viability, whereas both compounds exhibited appreciable cytotoxicity at 50 μM (Melis et al. 2014). In addition, CFTRinh-172 and GlyH-101 were shown to rapidly increase reactive oxygen species production and reduce mitochondrial membrane potential in both CFTR-expressing and CFTR-deficient cell lines. They also decreased cellular oxygen consumption, indicating CFTR-independent off-target effects on mitochondrial function (Kelly et al. 2010). These findings emphasize the need to carefully control inhibitor concentration and exposure duration in experimental and potential therapeutic applications. Additionally, intrinsic physicochemical limitations, such as poor water solubility, rapid clearance by intestinal washout, and reduced activity at physiologically relevant epithelial membrane potentials, further restrict their application (Sonawane et al. 2005; Thiagarajah et al. 2014).

Despite these limitations, CFTRinh-172 and GlyH-101 remain highly valuable tools for mechanistic studies when dosing and exposure duration are strictly controlled. In mouse models, the effective doses are well below the threshold for systemic toxicity; repeated low-dose administration does not affect feeding or drinking behavior, nor does it cause abnormalities in serum electrolytes, liver and kidney function, or peripheral blood cell counts (Ma et al. 2002; Sonawane et al. 2005, 2006). Both compounds remain valuable in elucidating CFTR-dependent secretion mechanisms; however, for clinical translation, there is an urgent need to improve target selectivity, enhance intestinal retention, and optimize local delivery strategies. Although crofelemer does not specifically target BEST4⁺ cells, its dual inhibitory activity against CFTR and CaCCs has made it a more clinically viable example. Its clinical approval not only validates the feasibility of targeting epithelial chloride secretion pathways but also highlights the critical importance of localized intestinal action and minimized systemic exposure in the development of such drugs.

ADRA2A represents a genetically supported, BEST4⁺-selective therapeutic target. It is the only adrenergic receptor subtype reported to be robustly expressed in the human intestinal epithelium and is highly enriched in BEST4⁺ cells. Norepinephrine-mediated ADRA2A activation suppresses cAMP-dependent ion secretion and reverses CTX-induced swelling in human BEST4⁺-enriched intestinal organoids (Wang et al. 2025), demonstrating on-target functional rescue in a pathophysiologically relevant model. While still preclinical, ADRA2A agonism offers a unique opportunity for cell-type-specific intervention, bypassing broad pathway inhibition and minimizing off-target effects. Further development will require pharmacokinetic optimization, selectivity profiling against other adrenergic subtypes, and rigorous in vivo validation of antisecretory efficacy and barrier-sparing effects.

### BEST4⁺ cells and feed additives for preventing diarrhea

At present, most feed additives have not been shown to specifically target BEST4⁺ cells. Nevertheless, they may influence the secretory and barrier microenvironment in which BEST4⁺ cells operate by enhancing barrier integrity, suppressing excessive secretion, modulating the microbiota, or attenuating inflammation. For example, combinations of plant essential oils containing carvacrol, cinnamaldehyde, and thymol, as well as monobutyrin and high-dose zinc oxide, have shown potential to alleviate piglet diarrhea, improve intestinal health during weaning, and strengthen barrier function (Rebucci et al. 2022; Stas et al. 2025; Yi et al. 2023; Zhao et al. 2023). It should be noted, however, that although high-dose zinc oxide has long been used to control post-weaning diarrhea in piglets, environmental and regulatory concerns about its use have become increasingly prominent (Hong et al. 2025; Peters et al. 2024).

**Fig. 4:**
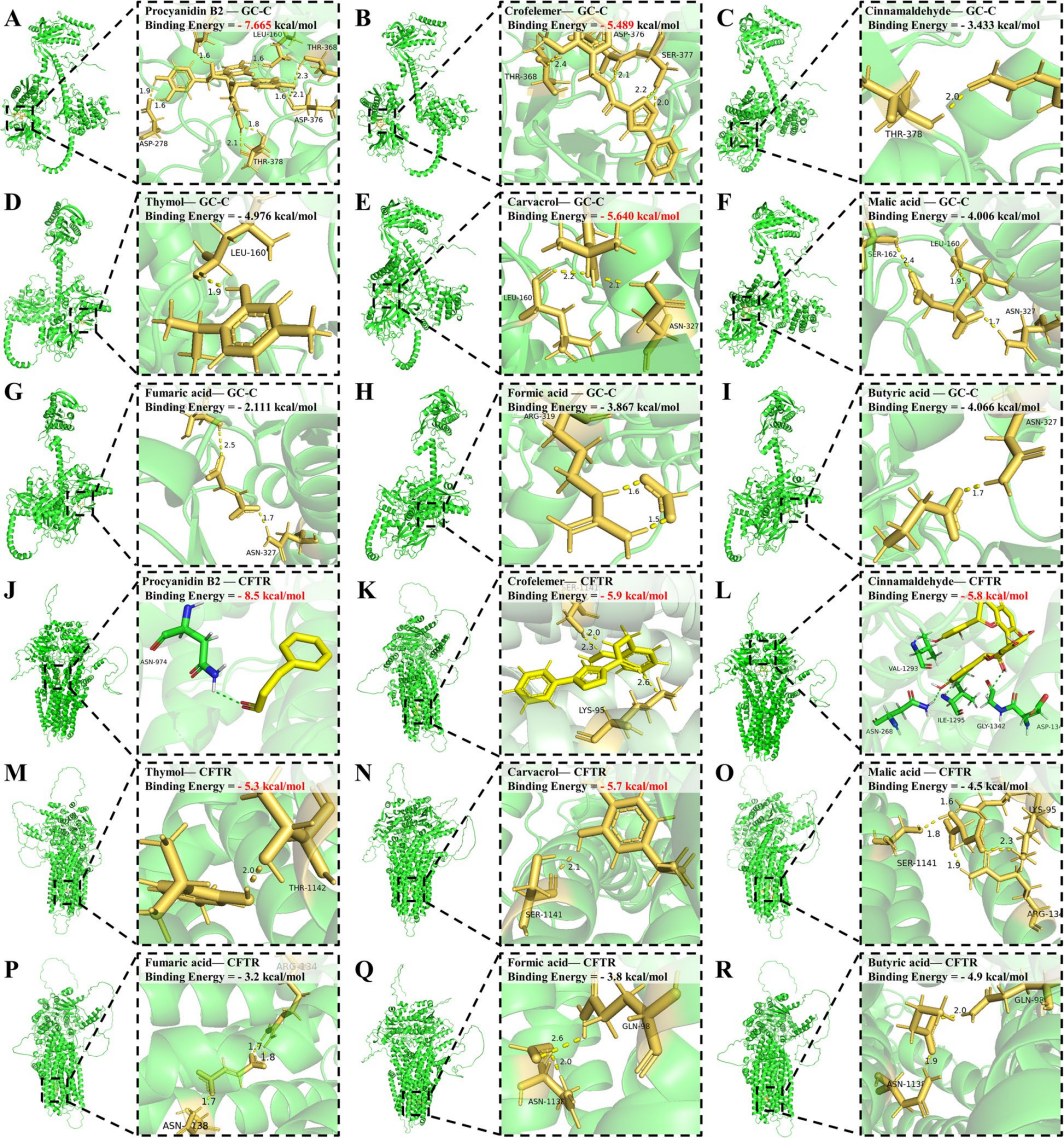
Molecular docking of representative candidate compounds with GC-C and CFTR. The figure shows the predicted binding poses and local interaction patterns of representative candidate compounds docked to GC-C and CFTR. Panels A–I show docking results for GC-C, including procyanidin B2 (**A**), crofelemer (**B**), cinnamaldehyde (**C**), thymol (**D**), carvacrol (**E**), malic acid (**F**), fumaric acid (**G**), formic acid (**H**), and butyric acid (**I**). Panels J–R show docking results for CFTR, including procyanidin B2 (**J**), crofelemer (**K**), cinnamaldehyde (**L**), thymol (**M**), carvacrol (**N**), malic acid (**O**), fumaric acid (**P**), formic acid (**Q**), and butyric acid (**R**). For each panel, the overall docking pose and the enlarged binding pocket are shown, together with the predicted binding energy. Binding energies highlighted in red indicate values lower than − 5.0 kcal/mol. These results provide preliminary structural clues suggesting that some natural compounds may interact with GC-C or CFTR, but such predictions remain computational and require further validation using cell-based functional assays and in vivo studies

The relevance of these additives lies not in proven direct regulation of BEST4⁺ cells but in their potential to modulate key physiological processes in which BEST4⁺ cells participate, including fluid secretion, regulation of the luminal microenvironment, mucus barrier function, and post-infectious responses. They may therefore be regarded as candidate modulators of BEST4⁺ cell-associated functional axes. For several natural small molecules, our molecular docking analysis suggests potential interactions with GC-C or CFTR (Fig. 4), providing preliminary structural clues regarding their possible antisecretory or barrier-protective effects. However, these findings are predictive and require further experimental validation. Representative antidiarrheal agents and their relationships to BEST4⁺ cell-associated pathways are summarized in Table 3.

**Table 3:** Antidiarrheal agents and their mechanisms

Antidiarrheal agent	Species/experimental model	Mechanism of action
**A. Interventions targeting molecular components enriched in BEST4⁺ cells**		
SSP2518	Human intestinal organoids; pigs	Small-molecule GC-C inhibitor that blocks STa-induced cGMP generation and downstream CFTR-dependent fluid secretion (Bijvelds et al. 2015; van Vugt et al. 2021)
Peptide 3	Human T84 cells	STa-derived competitive antagonist at the extracellular GC-C ligand-binding domain, preventing STa-triggered cGMP signaling (Tian et al. 2008)
Clonidine	Humans; rats	α₂-Adrenergic receptor agonist that promotes intestinal fluid absorption and reduces cAMP-dependent secretion; its relevance to BEST4⁺ cells reflects ADRA2A enrichment (Schiller et al. 1985; Nylander et al. 2022; Wang et al. 2025)
**B. Broader modulators of BEST4⁺ cell-associated ion-secretory pathways without cell-type specificity**		
CFTRinh-172	Mice	Thiazolidinone CFTR inhibitor that suppresses CFTR-mediated Cl⁻ secretion and CTX-induced intestinal fluid accumulation (Ma et al. 2002; Gao et al. 2024)
GlyH-101	Mice; human T84 cells	Pore-blocking CFTR inhibitor that reduces cAMP-stimulated Cl⁻ secretion and CTX-induced intestinal fluid accumulation (Muanprasat et al. 2004)
Crofelemer	Humans; human T84 cells	Proanthocyanidin that inhibits both CFTR- and TMEM16A/CaCC-mediated Cl⁻ secretion (Tradtrantip et al. 2010; Thiagarajah et al. 2014)
Procyanidins	Human T84 cells	Polyphenolic procyanidins that suppress CFTR-mediated Cl⁻ secretion in intestinal epithelial cells; docking suggests a possible direct interaction with CFTR (Schuier et al. 2005)
Cinnamaldehyde	Pigs	TRPA1 agonist that modulates epithelial ion and HCO₃⁻ transport in colonic mucosa; docking indicates a putative CFTR-binding site (Manneck et al. 2021)
**C. Conventional or supportive antidiarrheal agents without a demonstrated direct relationship to BEST4⁺ cells**		
Racecadotril	Humans	Prodrug converted to the enkephalinase inhibitor thiorphan, enhancing endogenous enkephalins at δ-opioid receptors and thereby reducing cAMP-dependent secretion and CFTR-mediated Cl⁻/water loss (Cézard et al. 2001; Fischbach et al. 2016)
Loperamide	Humans	Peripherally acting μ-opioid receptor agonist that diminishes intestinal propulsive motility and prolongs transit, with additional antisecretory effects under strongly stimulated conditions (Schiller et al. 1984)
Smectite	Human Caco-2 cells	A layered aluminosilicate clay that adsorbs water, toxins, viruses, and bile salts while strengthening the mucus layer and epithelial barrier to dampen secretory and inflammatory stimuli (Dupont and Vernisse 2009)
High-dose zinc oxide	Pigs	Pharmacological doses of ZnO release Zn^2^⁺, which can restrict enterotoxigenic bacterial growth and adhesion and improve tight-junction–dependent barrier function (Højberg et al. 2005; Tang et al. 2024)
Bismuth subsalicylate	Humans	A colloidal bismuth compound that coats the intestinal mucosa and binds toxins, with a salicylate component that reduces prostaglandin-mediated secretion and inflammation (Steinhoff et al. 1980)

## Challenges and future perspectives

BEST4⁺ cells are a rare but functionally important intestinal epithelial population involved in ion transport, luminal pH regulation, mucus hydration, and secretory responses. Their enrichment in CFTR, GC-C, OTOP2, CA7, and BEST4 suggests that they may serve as key epithelial effectors linking fluid-electrolyte homeostasis to secretory diarrhea, particularly in enterotoxin-associated diseases relevant to both human medicine and piglet production.

Although our understanding of BEST4⁺ cells has advanced substantially, several challenges remain. First, their lineage identity has not been fully resolved, and cell fate programs may differ across species. Second, current mechanistic evidence is still largely based on organoid models, and many proposed functions are inferred from spatial localization and molecular features rather than direct functional validation. A major limitation is the lack of suitable in vivo mouse models because the murine *Best4-ps* locus is annotated as a pseudogene; this limitation underscores the need for alternative animal models with conserved BEST4⁺ cell populations.

Future studies should therefore prioritize porcine and rat models together with human intestinal organoids. They should integrate single-cell omics, spatial transcriptomics, lineage-specific functional analyses, and disease-challenge experiments to clarify BEST4⁺ cell identity and determine how these cells regulate fluid secretion and barrier homeostasis under disease conditions relevant to both human medicine and livestock production. Overall, BEST4⁺ cells provide a new cellular framework for understanding intestinal secretion, diarrheal mechanisms, and other intestinal diseases.

## Data Availability

Not applicable.
